# SImplification of Medications Prescribed to Long-tErm care Residents (SIMPLER): study protocol for a cluster randomised controlled trial

**DOI:** 10.1186/s13063-017-2417-2

**Published:** 2018-01-12

**Authors:** Janet K. Sluggett, Esa Y. H. Chen, Jenni Ilomäki, Megan Corlis, Sarah N. Hilmer, Jan Van Emden, Choon Ean Ooi, Kim-Huong Nguyen, Tracy Comans, Michelle Hogan, Tessa Caporale, Susan Edwards, Lyntara Quirke, Allan Patching, J. Simon Bell

**Affiliations:** 1 Centre for Medicine Use and Safety, Faculty of Pharmacy and Pharmaceutical Sciences, Monash University, 381 Royal Parade, Parkville, Victoria 3052 Australia; 2 NHMRC Cognitive Decline Partnership Centre, Hornsby Ku-ring-gai Hospital, Hornsby, New South Wales Australia; 3 Department of Epidemiology and Preventive Medicine, Monash University, Melbourne, Victoria Australia; 4 Helping Hand Aged Care, North Adelaide, South Australia Australia; 5 Kolling Institute of Medical Research, Royal North Shore Hospital, Northern Clinical School, School of Medicine, University of Sydney, New South Wales, Australia; 6 Menzies Health Institute Queensland, Griffith University, Meadowbrook, Queensland Australia; 7 Centre for Health Services Research, The University of Queensland, Woolloogabba, Queensland Australia; 8 Drug & Therapeutics Information Service, Repatriation General Hospital, Daw Park, South Australia Australia; 9 Consumer representative, Alzheimer’s Australia, Scullin, Australian Capital Territory Australia; 10 Helping Hand Consumer and Carer Reference Group, Helping Hand Aged Care, North Adelaide, South Australia Australia; 11 Sansom Institute, School of Pharmacy and Medical Sciences, University of South Australia, Adelaide, South Australia Australia

**Keywords:** Medication regimen simplification, Residential aged care, Nursing Homes, Long-term care, Pharmacist, Medication administration, Randomised controlled trial

## Abstract

**Background:**

Complex medication regimens are highly prevalent in residential aged care facilities (RACFs). Strategies to reduce unnecessary complexity may be valuable because complex medication regimens can be burdensome for residents and are costly in terms of nursing time. The aim of this study is to investigate application of a structured process to simplify medication administration in RACFs.

**Methods:**

SImplification of Medications Prescribed to Long-tErm care Residents (SIMPLER) is a non-blinded, matched-pair, cluster randomised controlled trial of a single multidisciplinary intervention to simplify medication regimens. Trained study nurses will recruit English-speaking, permanent residents from eight South Australian RACFs. Medications taken by residents in the intervention arm will be assessed once using a structured tool (the Medication Regimen Simplification Guide for Residential Aged CarE) to identify opportunities to reduce medication regimen complexity (e.g. by administering medications at the same time, or through the use of longer-acting or combination formulations). Residents in the comparison group will receive routine care. Participants will be followed for up to 36 months after study entry. The primary outcome measure will be the total number of charted medication administration times at 4 months after study entry. Secondary outcome measures will include time spent administering medications, medication incidents, resident satisfaction, quality of life, falls, hospitalisation and mortality. Individual-level analyses that account for clustering will be undertaken to determine the impact of the intervention on the study outcomes.

**Discussion:**

Ethical approval has been obtained from the Monash University Human Research Ethics Committee and the aged care provider organisation. Research findings will be disseminated through conference presentations and peer-reviewed publications. SIMPLER will enable an improved understanding of the burden of medication use in RACFs and quantify the impact of regimen simplification on a range of outcomes important to residents and care providers.

**Trial registration:**

Australian New Zealand Clinical Trials Registry, ACTRN12617001060336. Retrospectively registered on 20 July 2017.

**Electronic supplementary material:**

The online version of this article (10.1186/s13063-017-2417-2) contains supplementary material, which is available to authorized users.

## Background

Residents are increasingly older, frailer and have more complex care needs when first entering residential aged care [1]. Polypharmacy is also highly prevalent, with a recent systematic review demonstrating that up to three-quarters of all residents take nine or more medications on a regular basis [2]. While polypharmacy is an important contributor to medication regimen complexity, other factors, such as multiple administration times, use of non-oral formulations, and specific administration instructions relating to crushing or administering medications with food all increase the overall complexity of a medication regimen [3].

Residents of aged care facilities have more complex medication regimens than their community-dwelling counterparts [4]. Because the majority of residents are dependent on facility staff for medication administration [1], complex medication regimens are costly for aged care providers in terms of nursing time. A time–motion observational study of 12 morning medication rounds within one Australian residential aged care facility (RACF), involving seven nurses across two high care units, reported that nursing staff spent an average of three hours administering medications to 35 residents during the morning medication round [5]. Complex medication regimens can also be burdensome for residents, particularly for residents with dementia. Strategies to reduce unnecessary medication regimen complexity are therefore likely to be important for both residents and aged care providers, particularly as increasing medication regimen complexity has been associated with a greater risk of hospitalisation among residents of aged care facilities [6].

Recent studies have focused on deprescribing as a strategy to reduce unnecessary medication regimen complexity [7]. Several randomised controlled trials have incorporated deprescribing interventions in RACFs with the aim of reducing polypharmacy [8]. An alternative approach that has received considerably less attention is to consolidate the number of medication administration times through such strategies as administering medications at the same time, standardising routes of administration, using long-acting formulations in preference to shorter-acting agents, and switching from multiple single-ingredient preparations to a combination formulation, where possible. Interventions to reduce medication regimen complexity have been successfully implemented in the hospital setting. In one controlled trial involving patients taking at least five medications on admission to a hospital in the United States (USA), delivery of a visual medication grid to physicians caring for patients in the intervention arm led to a significant reduction in both the number of medications and number of doses per day in comparison with controls [9]. Similarly, results of a pre–post study conducted in one Australian hospital showed that after clinical pharmacists received training to minimise medication regimen complexity, inpatients receiving a medication review had a smaller increase in regimen complexity at discharge than patients reviewed before the educational session [10].

Simplification of medication regimens in RACFs may offer such benefits as improved quality of life, particularly for residents with dementia or swallowing difficulties, those who regularly refuse to take medications, or residents who wish to participate in activities during the day or outside the aged care facility. One study involving two RACFs in the USA estimated that switching from a medication administered three times per day to a medication administered once daily for one resident, resulting in only one administration time per day, could save 63 min of nursing time per resident every month [11]. This suggests that medication regimen simplification may reduce the time needed for RACF staff to administer medications, thus freeing them to undertake other clinical duties. The primary aim of our study is to investigate application of a structured process to simplify medication administration for residents of aged care facilities.

## Methods

### Design and setting

SImplification of Medications Prescribed to Long-tErm care Residents (SIMPLER) is a non-blinded, matched-pair, cluster randomised controlled trial of a single multidisciplinary intervention to simplify medication regimens using a structured tool. The cluster design was chosen to minimise the potential for contamination associated with nurses and general medical practitioners providing care for a number of residents in the same facility.

Residents will be recruited from eight RACFs between April 2017 and October 2017. Details of participating RACFs can be obtained from the Australian New Zealand Clinical Trials Registry website. Recognising that the average length of stay for permanent residents of aged care facilities in Australia is 35 months [12], participants will be followed for up to 3 years after study entry. In Australia, RACFs (synonymous with long-term care facilities or nursing homes in other countries) provide supported accommodation for people with care needs that cannot be met in their own homes [1]. The RACFs participating in the SIMPLER study predominately cater for residents with high care needs, and are located in metropolitan and rural regions of South Australia. South Australia is the fourth largest state in Australia; it has a population of 1.723 million people [13].

This study is registered with the Australian New Zealand Clinical Trials Registry (ACTRN12617001060336). Details of the World Health Organization (WHO) trial registration data set for the SIMPLER study are provided in Additional file 1. The SPIRIT (Standard Protocol Items: Recommendations for Interventional Trials) checklist for the SIMPLER study is provided in Additional file 2.

### Governance and consultation

This study will be overseen by a project governance committee comprising the lead investigators and nominated representatives from the aged care provider organisation. The project governance committee will monitor protocol compliance, data collection and analysis throughout the trial. A stakeholder reference group comprising health care professionals, facility staff, consumers and carers will also provide ongoing input into the study. Membership of this group will be determined by consultation between the lead investigators and the aged care provider organisation. The investigators will work closely with the aged care provider organisation to conduct face-to-face consultations with relevant stakeholders at participating facilities during the pre-intervention period. Site-specific enablers and barriers to the delivery of the intervention will be identified and addressed.

### Participants

All permanent residents who are English-speaking and taking at least one regular medication will be eligible to participate. Residents estimated by RACF staff to have less than 3 months to live and those deemed by facility staff to be medically unstable (e.g. experiencing delirium) will be excluded. Residents may also be excluded at the discretion of RACF staff and their treating clinicians.

### Consent process

Residents who are eligible to participate in SIMPLER will be provided with oral and written information about the study. Residents will be invited to participate in the study by trained study nurses contracted by the aged care provider organisation and consent will be obtained from residents who have the capacity to consent to study involvement (Additional file 3). Consent to participate will be sought from the resident’s guardian, next of kin or significant other when the resident is unable to provide written informed consent to participate in the SIMPLER study. All information and consent materials will be written using the Dementia Australia Guide to Dementia Friendly Language [14]; study nurses will be trained using the Dementia Australia ‘Talk to Me’ tips for talking to people with dementia [15].

### Matching and randomisation

The SIMPLER study will recruit residents from five metropolitan RACFs and three rural RACFs. This includes four metropolitan RACFs with 90–110 beds, a larger metropolitan RACF with 155 beds, two rural RACFs with 40–50 beds each and a rural RACF with 100 beds (all bed numbers are approximate). The RACFs will be paired by the study investigators based on location (i.e. metropolitan or rural) and number of beds. The two smaller rural RACFs will be matched and the larger rural RACF will be matched with the closest metropolitan RACF of similar size. There will be approximately 10% difference in the total number of beds within each pair for two pairs, 20% difference for one pair and 40% difference in the total number of beds for the final pair, owing to the larger size of one metropolitan facility.

One RACF within each pair will be randomised to the intervention group and the other to the comparison (usual care) group. An independent pharmacoepidemiologist will perform the randomisation using the computerised random number generator within SAS (SAS Institute, Cary, NC). Study nurses, residents and staff at each RACF will be blinded to the allocation at the time of resident recruitment; however, owing to the nature of the intervention, it will not be possible to conceal allocation to the intervention or comparison group throughout the study period.

### Intervention

The Medication Regimen Simplification Guide for Residential Aged CarE (MRS GRACE) will be used to identify opportunities for simplifying medication regimens for SIMPLER participants in the intervention facilities. MRS GRACE was developed in consultation with a multidisciplinary expert panel and validated by two clinical pharmacists who independently applied the tool to de-identified medication charts for 50 residents. This implicit tool directs the user to consider the following five questions when considering whether a resident’s medication regimen may be simplified:

1. Is there a resident-related factor that precludes simplification?
2. Is there a regulatory or safety imperative that precludes simplification?
3. Is simplification likely to result in any clinically significant drug–drug, drug–food or drug–time interactions?
4. Is there no alternative formulation available that can support less complex dosing?
5. Is simplification likely to result in any unintended consequences?

The intervention will be implemented by an experienced clinical pharmacist who is accredited to perform residential medication management reviews (RMMRs). The intervention will be delivered after recruitment and baseline data collection. The study pharmacist will review medication charts, medical diagnoses and other relevant details obtained from the medical record, and use MRS GRACE to identify opportunities to simplify the medication regimen for study participants in the intervention facilities. The study pharmacist will forward a structured one-page report outlining recommendations for medication regimen simplification to the residential services manager and clinical nurse consultant at the aged care facility, and to the resident’s general medical practitioner (GP). The study pharmacist will record the amount of time spent reviewing medications, generating recommendations and communicating with key stakeholders (e.g. the resident, the resident’s guardian, next of kin or significant other, facility staff and GP).

Recommendations related to changes in medication administration times will require approval by the residential services manager, clinical nurse consultant or GP prior to implementation. All other recommendations (e.g. changes in medication strength, formulation or active ingredient) will require approval of the GP prior to implementation. Whether or not these recommendations are actioned will be at the discretion of the residential services manager or clinical nurse consultant and the resident’s GP, respectively. It will be the responsibility of the residential services manager or clinical nurse consultant and the resident’s GP to ensure that the resident or the resident’s guardian, next of kin or significant other are involved in the decision making process at all steps. This process was supported by the aged care provider organisation’s research ethics review panel and metropolitan medication advisory committee.

### Usual care

Residents in the comparison RACFs will continue to receive routine care.

**Fig. 1:**
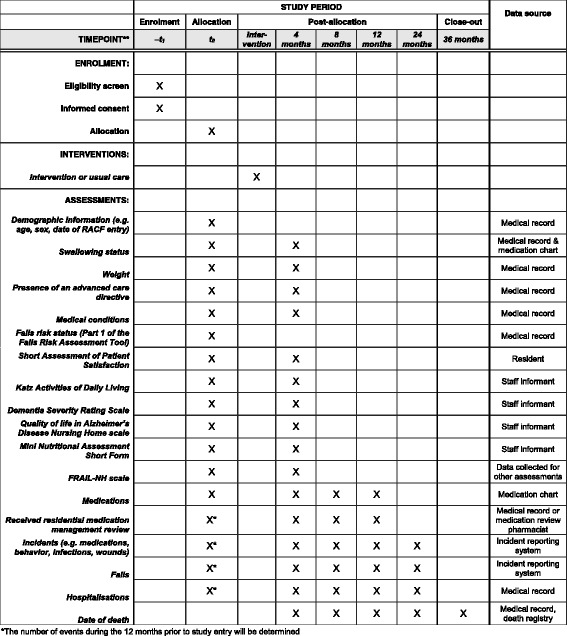
Schedule of enrolment, intervention and assessments for the SIMPLER study. RACF, residential aged care facility

All residents in the intervention and comparison RACFs will continue to be eligible to receive Australian Government-remunerated Quality Use of Medicines services and RMMRs during the study period. The Quality Use of Medicines service is provided by registered pharmacists; activities may include staff education, drug utilisation audits, contribution to medication advisory committee meetings and reviewing medication-related policies [16]. The RMMR process involves an accredited pharmacist providing a clinical medication review for residents of aged care facilities following a referral from the resident’s GP [16]. Although RMMRs and Quality Use of Medicines services may result in recommendations to cease or simplify medications, these services are not delivered with the specific aim of reducing regimen complexity. Data will be collected to determine whether residents received an RMMR in the 12 months prior to study entry, and during follow-up, as outlined in Fig. 1.

### Data collection and quality assurance

The SPIRIT Figure (Fig. 1) outlines the data to be collected for each participating resident at baseline, and at 4, 8, 12, 24 and 36 months after study entry. Study nurses contracted by the aged care provider organisation will be responsible for collecting data for each participating resident at baseline and 4 months after study entry.

The study nurses and pharmacist will undergo an orientation programme to become familiar with the aged care provider’s processes, policies and procedures. The study nurses will be trained in the consent process, data collection and use of the assessment tools. The study nurses and pharmacist will undergo mandatory police clearances for working with residents of aged care facilities.

Study nurses will collect demographic and diagnostic data from each resident’s medical record. Data relating to charted medications and medication use in the preceding 7 days will be extracted directly from each resident’s medication administration chart. Details of all prescription and non-prescription medications administered regularly and as required will be extracted. The medication name, strength, number of dosing times and administration details will be recorded.

Other clinical data will be collected via a series of validated assessments and recorded using a web-based electronic data extraction form on handheld tablets. The study nurses will seek input from a staff member who has known the resident for at least 2 weeks when undertaking staff-informant assessments. Concordance between the assessments performed by different study nurses will be checked for a 20% sample of participating residents from one RACF. A copy of the online data collection form is available from the authors on request.

If, when undertaking the study procedure, the study nurses or pharmacist identify any signs, symptoms or patterns of care that they believe require medical attention, this will be brought to the attention of the residential services manager or clinical nurse consultant at the relevant facility.

All other data will be entered into an electronic management system by research team members who are trained in data entry. To ensure accurate data entry by research team members, a second member of the research team will check data for the first 10 participants, and for a random 10% sample of records entered thereafter. Medications will be classified using the WHO Anatomical Therapeutic Chemical classification [17]. Reasons for hospitalisation will be coded according to the WHO International Statistical Classification of Diseases and Related Health Problems 10th Revision [18]. Enrolment, data collection, study conduct and any adverse outcomes will be monitored through weekly meetings with the research team, representatives of the aged care provider organisation, study nurses and pharmacist to ensure protocols are consistently implemented.

Data collected as part of this study will be treated confidentially and stored securely at the Centre for Medicine Use and Safety, Faculty of Pharmacy and Pharmaceutical Sciences, Monash University for a minimum of 15 years after publication of results in accordance with Monash University guidelines for medical research involving clinical trials. If electronic transfer between researchers is necessary, this will be performed using a secure transfer system and only de-identified information will be electronically transferred.

### Withdrawal from the study

Study investigators, the aged care provider organisation and staff at the relevant RACF will be notified if a resident is withdrawn from the study. Information that has been collected about the resident, prior to the withdrawal, will continue to be used in the data analysis. No new information will be collected or used after the resident has withdrawn from the study. The resident’s GP will continue to be responsible for reviewing the resident’s medications; the resident will continue to receive usual care from facility staff and other health professionals.

### Primary outcome measure

The primary outcome measure is the total number of charted medication administration times over a 24 hour period for regular medications at 4 months after study entry. This will be determined from medication data extracted by the study nurses. Short-term, nurse-initiated and *pro-re-nata* medications, and nutritional drinks will not be assessed. This outcome will also be determined at 8 and 12 months after study entry to assess the sustainability of the intervention.

### Secondary outcome measures

#### Time spent administering medications

The duration of time spent administering medications, based on the number of medications charted, administration times, dosage formulations and use of controlled medications or dose administration aids, will be estimated using data collected during a concurrent time–motion study and information abstracted from the medication administration charts at baseline and follow-up.

#### Medication incidents

Details of medication incidents occurring for participating residents will be determined from the electronic risk management database maintained by the aged care provider organisation. This database is used by facility staff to record the date of the medication incident, the type of incident (e.g. prescribing error, pharmacy dispensing error identified by facility staff, client error, administration error or adverse drug reaction), the severity and the resident involved.

#### Resident satisfaction

Residents will be interviewed by a study nurse using the revised version of the Short Assessment of Patient Satisfaction (SAPS) scale [19]. This seven-item scale is recommended in the Australian Government Dementia Outcomes Measurement Suite [20]. The SAPS scale has also been successfully used to assess satisfaction among residents of aged care facilities [21]. Residents who are unable to participate in this assessment are still eligible for inclusion in the study.

#### Quality of life

Quality of life will be assessed by a staff informant using the Quality of Life in Alzheimer’s Disease (QoL-AD) scale adapted for residents of aged care facilities. Each of the 15 measures included in the QoL-AD questionnaire is rated on a scale of one to four, with lower scores indicating reduced quality of life [22, 23]. The QoL-AD is one of the scales included in the Dementia Outcomes Measurement Suite [20] and has also been successfully used to assess quality of life among residents of aged care facilities with and without dementia [21].

#### Falls

Details of all falls for participating residents, including the date of the fall, severity and related health outcomes (e.g. fracture, hospitalisation) will be extracted from the electronic risk management database maintained by the aged care provider organisation. A fall will be defined as ‘an event that results in a person coming to rest inadvertently on the ground or floor or other lower level’ [24].

#### Hospitalisation

Overnight hospitalisations during the follow-up period will be determined from the electronic records maintained by the aged care provider organisation. Hospital admission and discharge dates, and the reasons for hospitalisation will be extracted.

#### Mortality

Deaths in the 36 months after the intervention will be determined from electronic RACF records and the Government of South Australia Consumer and Business Services: Births, Deaths and Marriages.

### Covariates

Data will be collected for each covariate using the following validated scales, to enable adjustment in statistical analyses for the SIMPLER study:

(i) *Patient demographics* including age, sex, weight, swallowing status, date of admission to the RACF, presence of an advanced care directive, and whether the resident self-administers their medication will be recorded.
(ii) *RMMR* data will be screened to determine the date an RMMR was provided.
(iii) *Medical conditions* will be extracted directly from each resident’s medical record. Charlson Comorbidity Index, a comorbidity index that assigns weights for specific medical conditions to measure disease burden and predict mortality, will be calculated for each participant [25, 26].
(iv) *Dementia severity* will be assessed for each resident using the 12-item Dementia Severity Rating Scale, which provides a validated measure of impairment across the major functional and cognitive domains affected in Alzheimer’s disease [27].
(v) *Falls risk status* will be determined using Part 1 of the Falls Risk Assessment Tool, which is widely used to assess falls risk in Australian RACFs [28].
(vi) *Activities of daily living* (ADL) will be assessed using the six-item Katz ADL scale [29].
(vii) *Nutritional status* will be assessed using the six-item Mini Nutritional Assessment Short Form, a validated tool for assessing malnutrition in older people [30].
(viii) *Frailty status* will be assessed using the seven-item FRAIL-NH scale [31, 32], which was adapted from the FRAIL screening test to screen for frailty in the residential aged care setting.
(ix) *Incidents* that are routinely collected by the aged care provider organisation (e.g. behaviour incidents, infections, wounds) will be recorded for participating residents.

### Analysis plan

#### Sample size calculation

A sample size calculator software package (Sample Size Calculator V2.0, Health Services Research Unit, University of Aberdeen) [33] was used to estimate the sample size needed to assess the primary outcome. Validation of the MRS GRACE tool by two clinical pharmacists using de-identified medication charts for 50 residents who were administered medications at least twice per day showed that the number of administration times could be reduced for 23 (46%) residents. As not all recommendations may be implemented by the residential services manager, clinical nurse consultant or GP, we anticipate that the number of administration times could be reduced for 25% of SIMPLER participants receiving the intervention. Allowing for a reduction in administration times for 5% of residents in the comparison group, and assuming 80% power, 5% significance and an intracluster correlation coefficient of 0.1 [34, 35], 22 residents would be required from each facility (i.e. 176 residents). Allowing for a 10% attrition of participants over 4 months, as residents estimated to have less than 3 months to live are not eligible to participate, the SIMPLER study will need to recruit a minimum of 194 residents.

#### Quantitative analyses

Individual-level analyses that account for clustering will be undertaken to determine the relationship between the intervention and study outcomes. Analyses will be undertaken using an intention-to-treat principle; however, sensitivity analyses will be undertaken among residents prescribed medications at least twice per day on a regular basis at baseline, and on a per-protocol basis, in which the intervention group will be restricted to residents for whom at least one medication simplification recommendation was implemented. Mixed models with RACF as a random effect will be utilised to analyse the association between intervention and outcomes. Models will be adjusted for resident characteristics at baseline (including number of medication administration times). Multiple imputation will be used to impute missing covariate values where necessary. Data will be analysed using SAS (SAS Institute, Cary, NC) and the Statistical Package for the Social Sciences (SPSS, Inc., Chicago, IL).

#### Economic analysis

A within-trial cost-effectiveness analysis will be conducted alongside the clinical trial to synthesis the costs and outcomes of SIMPLER. The primary outcome measure will be quality of life, measured using the QoL-AD instrument. The total cost associated with medication use and administration for participants in the intervention and comparison groups will be estimated at baseline and follow-up (at 4 and 8 months after study entry). Medication incidents, falls and hospitalisations will be incorporated in the economic analysis. Appropriate adjustment for the cluster design of the trial will be undertaken using multilevel models. The multilevel models approach can incorporate cluster level random effects by explicitly recognising clustering in parameter estimations while adjusting for cluster and individual covariates that may vary between groups and clusters [36]. Additionally, multilevel models can accommodate non-normal distribution of the error terms (e.g., gamma distribution) [37] and do not require strict asymptotic assumptions to be met [36], which provides more precise estimates than alternative approaches. The economic analysis will be conducted using Stata (College Station, TX: StataCorp LP).

### Dissemination

Manuscripts presenting study results will be submitted for presentation at relevant national and international conferences and submitted for publication in peer-reviewed scientific journals. Research findings will also be disseminated to consumers and carers, clinicians, researchers, aged care providers and policy makers through the National Health and Medical Research Council (NHMRC) Cognitive Decline Partnership Centre [38].

All RACFs participating in this research will be provided with a copy of a final report summarising key study findings. The final report will also be forwarded to all Helping Hand Medication Advisory Committees. Research participants and clinicians will be provided with a lay summary of findings. Research findings will be further disseminated to consumers, carers and clinicians at a public presentation. Consumers and carers will be invited to participate in the delivery of this presentation.

## Discussion

The SIMPLER study will lead to an improved understanding of the burden of medication administration in RACFs and quantify the impact of regimen simplification on a range of outcomes important to residents and care providers. The study is underpinned by extensive stakeholder engagement. This has assisted study investigators to identify barriers to participation and uptake of the recommendations for medication regimen simplification, and address these factors. The process of medication regimen simplification will involve the application of a structured tool (MRS GRACE), which has been validated in the residential aged care setting. The use of a cluster randomised controlled trial to randomise at the facility level, rather than the resident level, will reduce the risk that residential services managers, clinical nurse consultants and GPs will simplify medication regimens for all residents under their care, regardless of treatment group allocation.

Study participants will be recruited from a single aged care provider organisation, which means that study findings may not be generalisable to all older people. It is recognised that there may be differences in health status, cognitive function or involvement with medications between participating residents and those who decline to participate. The SIMPLER study nurses, residents and staff at each RACF will be blinded to treatment allocation at the time of recruitment to minimise the risk of selection bias. However, ascertainment bias is possible as some of the secondary outcomes are derived from assessments completed by residents (resident satisfaction) and staff informants (quality of life), who may be aware of the treatment allocation when the assessments are undertaken at the 4 month follow-up.

Considerable time is spent administering medications in RACFs and complex medication regimens have been linked to poor health outcomes for residents. It is recognised that some medication regimen complexity is unnecessary and could be reduced by consolidating dosing times. The SIMPLER study will use a structured approach to target unnecessary medication regimen complexity in RACFs, and generate evidence to inform clinical practice and policy decisions in this setting.

### Trial status

This manuscript presents version 1.4 (1 March 2017) of the SIMPLER protocol. The date of first participant enrolment was 24 April 2017 and recruitment is ongoing (anticipated end date 31 October 2017).

## Additional files

Additional file 1: WHO trial registration data set for the SIMPLER study. (PDF 327 kb)

Additional file 2: SPIRIT checklist for the SIMPLER trial protocol. (PDF 173 kb)

Additional file 3: Model consent form for the SIMPLER study. (PDF 293 kb)

## Abbreviations

ADL: Activities of daily living
GP: General medical practitioner
MRS GRACE: Medication Regimen Simplification Guide for Residential Aged CarE
NHMRC: National Health and Medical Research Council
QoL-AD: Quality of Life in Alzheimer’s Disease
RACF: Residential aged care facility
RMMR: Residential Medication Management Review
SAPS: Short Assessment of Patient Satisfaction
SIMPLER: SImplification of Medications Prescribed to Long-tErm care Residents
SPIRIT: Standard Protocol Items: Recommendations for Interventional Trials
WHO: World Health Organization
