# Supplementary material for: SImplification of Medications Prescribed to Long-tErm care Residents (SIMPLER): study protocol for a cluster randomised controlled trial
Source: Trials. 2018 Jan 12;19:37. doi: 10.1186/s13063-017-2417-2 (PMC5767065; doi:10.1186/s13063-017-2417-2)
Supplement: Supplementary file 1 — WHO trial registration data set for the SIMPLER study. (PDF 327 kb) [file 13063_2017_2417_MOESM1_ESM.pdf]

Additional file 1: Table S1: The World Health Organization (WHO) Trial Registration Data Set for the SIMPLER study.

Data category	Information
Primary Registry and Trial Identifying Number	Australian New Zealand Clinical Trials Registry<br>ACTRN12617001060336
Date of Registration in Primary Registry	20/07/2017
Secondary Identifying Numbers	Universal Trial Number: U1111-1199-0894
Source(s) of Monetary or Material Support	National Health and Medical Research Council (NHMRC) Partnership Centre for Dealing with Cognitive and Related Functional Decline in Older People (the Cognitive Decline Partnership Centre or CDPC)
Primary Sponsor	Centre for Medicine Use and Safety, Faculty of Pharmacy and Pharmaceutical Sciences, Monash University<br>381 Royal Parade, Parkville VIC 3052, Australia
Secondary Sponsor(s)	None
Contact for Public Queries	Dr Janet Sluggett<br>Centre for Medicine Use and Safety, Faculty of Pharmacy and Pharmaceutical Sciences, Monash University<br>381 Royal Parade, Parkville VIC 3052, Australia<br>Phone: +61 3 99039533<br>Email: janet.sluggett@monash.edu
Contact for Scientific Queries	Dr Janet Sluggett<br>Centre for Medicine Use and Safety, Faculty of Pharmacy and Pharmaceutical Sciences, Monash University<br>381 Royal Parade, Parkville VIC 3052, Australia<br>Phone: +61 3 99039533<br>Email: janet.sluggett@monash.edu
Public Title	SImplification of Medications Prescribed to Long tErm care Residents (SIMPLER)
Scientific Title	SImplification of Medications Prescribed to Long tErm care Residents (SIMPLER): a cluster randomised trial of a structured process to reduce unnecessary medication regimen complexity

Countries of Recruitment	Australia
Health Condition(s) or Problem(s) Studied	Polypharmacy, Medication incidents, Dementia, Cognitive impairment, Falls, Hospitalisation, Frailty
Intervention(s)	Medications taken by residents in the intervention arm will be assessed once using a structured tool to identify opportunities to reduce medication regimen complexity. Residents in the comparison group will receive routine care.
Key Inclusion and Exclusion Criteria	Key Inclusion Criteria: Permanent residents of aged care facilities who are aged ≥18 years, English-speaking and taking at least one medication.<br>Key Exclusion Criteria: Residents estimated by RACF staff to have less than three months to live and those deemed by facility staff to be medically unstable (e.g. experiencing delirium) will be excluded. Residents may also be excluded at the discretion of RACF staff and their treating clinicians.<br>English-speaking residents who are unable to participate in the Short Assessment of Patient Satisfaction (SAPS) are still eligible for inclusion in the study.
Study Type	Study type: Interventional<br>Study design: Matched-pair, cluster randomised controlled trial<br>Masking: Open (masking not used)<br>Intervention assignment: Parallel<br>Procedure for enrolling a subject and allocating the treatment (allocation concealment procedures): Study nurses, residents and staff at each RACF will be blinded to the allocation at the time of resident recruitment. Due to the nature of the intervention, it will not be possible to blind study nurses, residents, RACF staff, clinicians and study investigators to the intervention assignment throughout the study period.<br>Methods used to generate the sequence in which subjects will be randomised (sequence generation): Aged care facilities will be paired by the study investigators based on location (i.e. metropolitan or rural) and number of beds. One RACF within each pair will be randomised to the intervention group and the other to the

	comparison (usual care) group. An independent pharmacoepidemiologist will perform the randomisation using the computerised random number generator within SAS (SAS Institute, Cary, NC). Block randomisation will be used to ensure an equal number of intervention and comparison groups.
Date of First Enrollment	24/04/2017
Target Sample Size	194 residents
Recruitment Status	Recruiting
Primary Outcome(s)	Primary Outcome: Total number of charted medication administration times over a 24 hour period for regular medications.<br>Metric/method of measurement: Determined from medication data extracted by the study nurses.<br>Timepoint: Baseline, and at 4 months after study entry.
Key Secondary Outcomes	Secondary Outcome 1: Total number of charted medication. administration times over a 24 hour period for regular medications.<br>Metric/method of measurement: Determined from medication data extracted by the study nurses.<br>Timepoint: 8 and 12 months after study entry, to assess the sustainability of the intervention.<br><br>Secondary outcome 2: Duration of time spent administering medications.<br>Metric/method of measurement: Determined from medication data extracted by the study nurses and from data collected during a concurrent time-motion study.<br>Timepoint: Baseline, and at 4 and 8 months after study entry.<br><br>Secondary outcome 3: Costs associated with medication administration.<br>Metric/method of measurement: Determined from medication data extracted by the study nurses and from data collected during a concurrent time-motion study.<br>Timepoint: Baseline, and at 4 and 8 months after study entry.

	Secondary outcome 4: Resident satisfaction.<br>Metric/method of measurement: Assessed using the 7-item revised version of the Short Assessment of Patient Satisfaction (SAPS) scale.<br>Timepoint: Baseline, and at 4 months after study entry.<br><br>Secondary outcome 5: Quality of life.<br>Metric/method of measurement: Assessed by a staff informant using the 15-item Quality of Life in Alzheimer's Disease (QoL-AD) scale adapted for residents of aged care facilities.<br>Timepoint: Baseline, and at 4 months after study entry.<br><br>Secondary outcome 6: Change in medication incidents (e.g. prescribing errors, pharmacy dispensing errors identified by facility staff, client errors, administration errors or adverse drug reactions).<br>Metric/method of measurement: Determined from the electronic records maintained by the aged care provider organisation.<br>Timepoint: Baseline, and at 4, 8, 12 and 24 months after study entry.<br><br>Secondary outcome: Number of falls.<br>Metric/method of measurement: Determined from the electronic records maintained by the aged care provider organisation.<br>Timepoint: Baseline, and at 4, 8, 12 and 24 months after study entry.<br><br>Secondary outcome 8: All-cause overnight hospitalisations.<br>Metric/method of measurement: Determined from the electronic records maintained by the aged care provider organisation.<br>Timepoint: Baseline, and at 4, 8, 12 and 24 months after study entry.<br><br>Secondary outcome 9: All-cause mortality.<br>Metric/method of measurement: Determined from the electronic records maintained by the aged care provider organisation and/or records maintained by the Government of South Australia Consumer and Business Services: Births, Deaths and Marriages.

	Timepoint: Baseline, and at 4, 8, 12, 24 and 36 months after study entry.
