# Supplementary material for: SImplification of Medications Prescribed to Long-tErm care Residents (SIMPLER): study protocol for a cluster randomised controlled trial
Source: Trials. 2018 Jan 12;19:37. doi: 10.1186/s13063-017-2417-2 (PMC5767065; doi:10.1186/s13063-017-2417-2)
Supplement: Supplementary file 3 — Model consent form for the SIMPLER study. (PDF 293 kb) [file 13063_2017_2417_MOESM3_ESM.pdf]

Letterhead

CONSENT FOR RESEARCH PARTICIPANTS

SImplification of **M**edications **P**rescribed to **L**ong t**E**rm care **R**esidents **(SIMPLER)**

I have been invited to participate in this research project.

**I understand that:**	Yes	No
1. I may not directly benefit from taking part in the research project.	☐	☐
2. While information gained during the research project may be published, I will not be identified and my personal results will remain confidential.	☐	☐
3. I can withdraw from the research project at any stage and this will not affect my medical care, now or in the future. I understand that any information provided prior to my withdrawal will continue to be used in the data analysis.	☐	☐
4. I have had the opportunity to discuss taking part in this research project with a family member or friend.	☐	☐

**I consent to the following:**	Yes	No
5. I have read and understood the Information Sheet provided to me, and I hereby consent to take part in this research project.	☐	☐
6. I give permission for the research team to contact my general practitioner, community pharmacy or the pharmacist providing residential medication management reviews (RMMRs) at the facility if further information is required for the purpose of this study.	☐	☐
7. I give permission for the research team to access data about my medications, RMMRs, medical conditions, clinical assessments, falls and other incidents, and any periods I am admitted to hospital, over the previous twelve months and during the study period.	☐	☐

**I consent to the following:**	Yes	No
8. I give permission for the research team to access data about me from the South Australian Ambulance Service	☐	☐
9. I give permission for the research team to access data about me from the South Australian Consumer and Business Service: Births, Deaths and Marriages during the study period.	☐	☐
10. I consent to be invited to provide feedback on the impact of this research project.	☐	☐

Participant name : ______________________________

Participant signature : ______________________________

Dated : ______________________________

I certify that I have explained the research project to the participant and consider that he/she understands what is involved.

Research Nurse signature : ______________________________

Dated : ______________________________

Witness signature : ______________________________

Dated : ______________________________
